# Reprogramming of Lipid Metabolism as a New Driving Force Behind Tauroursodeoxycholic Acid-Induced Neural Stem Cell Proliferation

**DOI:** 10.3389/fcell.2020.00335

**Published:** 2020-05-26

**Authors:** Marta B. Fernandes, Márcia Costa, Maria Filipe Ribeiro, Sónia Siquenique, Sónia Sá Santos, Joana Martins, Ana V. Coelho, Margarida F. B. Silva, Cecília M. P. Rodrigues, Susana Solá

**Affiliations:** ^1^Research Institute for Medicines (iMed.ULisboa), Faculty of Pharmacy, Universidade de Lisboa, Lisbon, Portugal; ^2^Instituto de Tecnologia Química e Biológica António Xavier, Universidade Nova de Lisboa, Lisbon, Portugal

**Keywords:** metabolism, mitochondria, neural stem cells, proliferation, tauroursodeoxycholic acid

## Abstract

Recent evidence suggests that neural stem cell (NSC) fate is highly dependent on mitochondrial bioenergetics. Tauroursodeoxycholic acid (TUDCA), an endogenous neuroprotective bile acid and a metabolic regulator, stimulates NSC proliferation and enhances adult NSC pool *in vitro* and *in vivo*. In this study, we dissected the mechanism triggered by this proliferation-inducing molecule, namely in mediating metabolic reprogramming. Liquid chromatography coupled with mass spectrometry (LC-MS) based detection of differential proteomics revealed that TUDCA reduces the mitochondrial levels of the long-chain acyl-CoA dehydrogenase (LCAD), an enzyme crucial for β-oxidation of long-chain fatty acids (FA). TUDCA impact on NSC mitochondrial proteome was further confirmed, including in neurogenic regions of adult rats. We show that LCAD raises throughout NSC differentiation, while its silencing promotes NSC proliferation. In contrast, nuclear levels of sterol regulatory element-binding protein (SREBP-1), a major transcription factor of lipid biosynthesis, changes in the opposite manner of LCAD, being upregulated by TUDCA. In addition, alterations in some metabolic intermediates, such as palmitic acid, also supported the TUDCA-induced *de novo* lipogenesis. More interestingly, a metabolic shift from FA to glucose catabolism appears to occur in TUDCA-treated NSCs, since mitochondrial levels of pyruvate dehydrogenase E1-α (PDHE1-α) were significant enhanced by TUDCA. At last, the mitochondria-nucleus translocation of PDHE1-α was potentiated by TUDCA, associated with an increase of H3-histones and acetylated forms. In conclusion, TUDCA-induced proliferation of NSCs involves metabolic plasticity and mitochondria-nucleus crosstalk, in which nuclear PDHE1-α might be required to assure pyruvate-derived acetyl-CoA for histone acetylation and NSC cycle progression.

## Graphical Abstract

TUDCA induces a metabolic shift to boost NSC proliferation. The FA and glucose oxidation counterbalance are crucial for NSC fate regulation. In early-differentiating NSCs, TUDCA increases *de novo* lipogenesis and proliferation by inducing a metabolic shift from FA to glucose catabolism that facilitates NSC cell cycle-associated H3 acetylation.

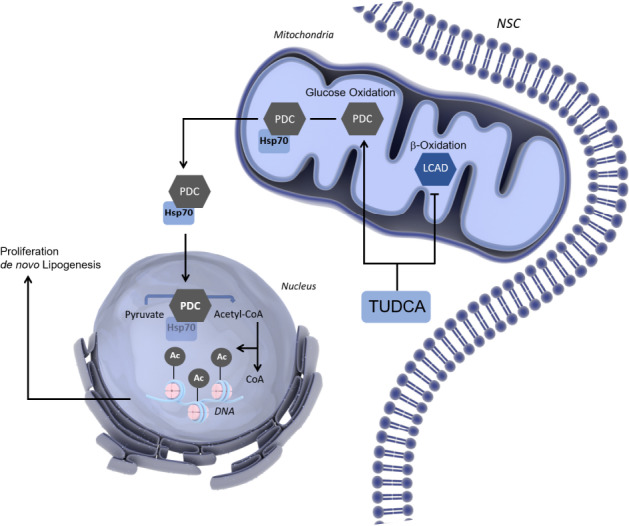

## Introduction

Over the past few years, our perception of neural stem cell (NSC) potential has greatly increased, although we are only beginning to understand their metabolic profile in physiological and pathological context (Ottoboni et al., 2017). A more comprehensive understanding of how adult NSCs rely on different metabolic pathways to keep up with cell-specific bioenergetic demands will certainly contribute to tune NSCs toward the desired response, including when therapeutically addressing aging and complex metabolic and neurodegenerative diseases (Wallace, 2005; Folmes et al., 2013; Knobloch and Jessberger, 2017).

Mitochondrial dynamics and bioenergetics are closely associated to NSC fate and behavior (Kann and Kovács, 2007; Wanet et al., 2015; Xavier et al., 2015). In this regard, mitochondrial dysfunction can be an underlying problem in the depletion of the stem cell pool and impaired neurogenesis (Wallace, 2005; Khacho et al., 2017). Mitochondria are also responsible for long-term survival, differentiation and synaptic integration of newborn neural cells (Xavier et al., 2015). Therefore, mitochondria and its regulatory network have major implications toward a more efficient use of neural regeneration therapies (Casarosa et al., 2014).

Increasing evidence suggests that metabolic plasticity is crucial to the transition between stemness maintenance and lineage specification (Folmes et al., 2013; Knobloch and Jessberger, 2017). Metabolic changes between stem cells and their progeny also suggest that mitochondrial mass and activity increase with lineage progression to meet the robust energy demands associated with differentiation (Wanet et al., 2015; Hu et al., 2016). Thus, the identity of stage-specific metabolic programs and their impact on adult neurogenesis need to be explored as we are now starting to unravel mitochondria molecular adaptations of metabolic circuits under this scenario. On the road of cellular metabolic pathways, lipid metabolism has also been largely neglected for the role it may play in the neurogenesis process. However, lipids emerge in NSC life as building blocks of membranes, an alternative energy source and as signaling entities (Knobloch, 2016). Indeed, fatty acids (FAs) have been shown to be produced endogenously in adult NSCs and a novel mechanism governing adult neurogenesis has been identified, in which lipogenesis determines the proliferative activity of NSCs (Folmes et al., 2013). Interestingly, during the transition from quiescent to active NSCs, glycolysis and FA oxidation (FAO) gradually decrease, while dependence on glucose to supply oxidative phosphorylation (OXPHOS) for energy generation and lipogenesis for NSC proliferation tend to increase (Shin et al., 2015; Fidaleo et al., 2017).

Apart from signaling pathways responsible for mediating the NSC metabolic state, the redistribution of nuclear or mitochondrial proteins has also emerged as a novel direct way of interorganellar coordination (Lionaki et al., 2016). Surprisingly, one of the largest multiprotein complexes known, the mitochondrial pyruvate dehydrogenase complex (PDC), translocates to the nucleus of mammalian cells. In the nucleus, PDC was shown to be functional and to provide a novel pathway for nuclear acetyl-CoA synthesis in support of histone acetylation and epigenetic regulation (Sutendra et al., 2014). The recent knowledge on the metabolic switches ruling NSC transformation into immature neurons describe fateful metabolic shifts, controlling NSC identity (Knobloch and Jessberger, 2017). Therefore, specific modulation of metabolic pathways might be useful to improve adult neurogenesis.

Ursodeoxycholic acid (UDCA), an endogenous bile acid FDA-approved for the treatment of cholestatic liver diseases is used as a cytoprotective agent that strongly detain programmed cell death (Rodrigues et al., 1998a, b, 1999; Amaral et al., 2009; Vang et al., 2014). Tauroursodeoxycholic acid (TUDCA) is the taurine-conjugated form of UDCA. After conjugation with taurine, TUDCA is orally bioavailable and able to penetrate the CNS (Keene et al., 2002). TUDCA exhibits anti-inflammatory effects and was shown to attenuate neuronal loss in neurodegenerative diseases (Rodrigues et al., 2003; Nunes et al., 2012; Gronbeck et al., 2016). Importantly, gene expression microarray analysis demonstrated that TUDCA specifically modulates several enzymes involved in FA metabolism in primary rat hepatocytes (Castro et al., 2005). TUDCA was also shown to regulate energy metabolism, through the reduction of endoplasmic reticulum stress and improvement of impaired insulin signaling, leading to restored glucose homeostasis in obese and diabetic mice models (Ozcan et al., 2006; Guo et al., 2015). Moreover, it is known that this singular bile acid has an important role in neuroprotection and neurogenesis through mitochondrial regulation (Rodrigues et al., 2000; Xavier et al., 2014). TUDCA prevents early differentiation induced-mitochondrial alterations in a mouse NSC line, eliciting a marked increase in NSC population at S phase with a subsequent reduction at G1 phase, which is typical of cells in expansion. The resulting increase in NSC pool was shown to occur in a mitochondrial redox state and ATP-dependent manner (Xavier et al., 2014). Recently, the potential therapeutic effect of TUDCA in increasing the NSC pool and early choice toward neuronal differentiation *in vivo* was uncovered, reinforcing the proliferative and pro-neurogenic effects of this singular bile acid (Soares et al., 2018).

Here we sought to investigate the molecular mechanisms underlining TUDCA control of NCS proliferation and further exploit its potential effect in enhancing the NSC pool. Our results deciphered TUDCA-induced changes in mitochondrial proteomic signature of NSCs. Importantly, the downregulation of LCAD *in vitro* and *in vivo* appeared as a major finding, where the fine-tuning of mitochondrial long-chain FA β-oxidation in NSCs leads to important metabolic shifts in subcellular compartments.

## Materials and Methods

### Ethics Statement

The two mouse NSC lines, NS-TGFP and CGR8, used in this study were both obtained from Dr. Smith’s Laboratory, University of Cambridge, Cambridge, United Kingdom. The NS-TGFP line was provided by Dr. Henrique, University of Lisbon, Lisbon, Portugal and the CGR8 line was provided by Dr. Margarida Diogo, University of Lisbon, Lisbon, Portugal. The animal experiments were performed in accordance with Portuguese laws (DL 113/2013, 2880/2015, 260/2016) on Animal Care and with the EU Directive (86/609/EEC; 2010/63/EU) on the protection of animals used for experimental and other scientific purposes. In addition, animal welfare fulfilled the recommendation from the “Guide for the Care and Use of Laboratory Animals” prepared by the National Academy of Sciences and published by the National Institutes of Health (NIH publication 86–23 revised 1985). The Animal Ethical Committee at the Faculty of Pharmacy, University of Lisbon, Portugal waived the need for approval.

### Cell Culture

The CGR8 competent cell line was established from the inner cell mass of a 3.5-days male pre-implantation mouse embryo (ECACC 07032901) (Nichols et al., 1990; Smith, 1991; Mountford et al., 1994). Tau-GFP mouse NSC (NS-TGFP) cells were derived from 14.5-dpc mouse fetal forebrain, and constitutively express the fusion protein tau-GFP (Pratt et al., 2000; Silva et al., 2006). Mouse cell lines were established using a method that produces pure cultures of adherent bonafide NSCs, which continuously expand by symmetrical division and are both capable of tripotential differentiation (Conti et al., 2005; Pollard et al., 2006; Glaser et al., 2007). NSCs were grown in monolayer as previously described (Spiliotopoulos et al., 2009) and routinely maintained in undifferentiating medium (self-renewal conditions), Euromed-N medium (EuroClone S.p.A., Pavia, Italy), supplemented with 1% N-2 supplement (Gibco, Thermo Fisher Scientific, Inc., United States), 20 ng/μL epidermal growth factor (EGF; PeproTech EC, United Kingdom), 20 ng/μL basic fibroblast growth factor (bFGF; PeproTech EC) and 1% penicillin-streptomycin (Pen-Strep; Gibco, Thermo Fisher Scientific, Inc.), in 75 cm^2^ tissue culture (TC) treated flasks (Falcon, Corning Inc., New York, NY, United States) at 37°C in a humidified atmosphere of 5% CO_2_. Neural differentiation was performed by first plating NSCs in undifferentiating medium onto TC-treated cell culture dishes at 5.4 × 10^6^ cells/cm^2^ for 24 h. After 24 h of cell stabilization, the medium was changed to optimized neuronal differentiation-inducing medium, Euromed-N medium supplemented with 0.5% N-2 supplement, 1% B-27 supplement (Gibco, Thermo Fisher Scientific, Inc.), 10 ng/μL bFGF, and 1% Pen-Strep. For NSCs that remained undifferentiated, the medium was not changed. Differentiating NS-TGFP cells at 8 × 10^5^ cells/cm^2^ were at fixed at 0, 3, 6, 24, 48, and 72 h and processed for immunoblotting. For cellular treatments, cells were maintained in differentiating and undifferentiating medium for 24 h. Then, cells were collected and processed for immunoblotting, differentiated cells were also processed for Oil-Red-O staining, gas chromatography-mass spectrometry (GC-MS) analysis, immunocytochemistry, and quantitative RT-PCR (qRT-PCR).

### Animal Models and TUDCA Delivery

Mini-osmotic pumps (Alzet, Cupertino) were implanted in 6-weeks old male Wistar rats for intraventricular infusion of 300 μM TUDCA dissolved in artificial cerebrospinal fluid (aCSF) or aCSF alone, as vehicle, for continuous dosing of unrestrained laboratory animals. Cannulas were inserted in the lateral ventricle of the right hemisphere at the following coordinates: anterior-posterior: –0.4 mm, medial-lateral: 1.2 mm, dorso-ventral: 3.5 mm, having the bregma as a reference. The flow was continuous with a delivery rate was 0.25 μL/h for 28 days. After 28 days of treatment, animals were perfused with saline solution (NaCl 0.9%), and fixed with 4% paraformaldehyde (PFA) in PBS. Animals were sacrificed by decapitation and the brains immediately removed. Contralateral hemisphere was sectioned to perform immunohistochemistry assays.

### Cellular Treatments

Cells were treated with 100 μM of TUDCA (T0266; Sigma-Aldrich Corp.) after 24 h of plating and/or upon medium change for differentiation, and then collected after 24 h for protein extraction and other analyses.

### Total, Mitochondrial, Cytosolic, and Nuclear Protein Extraction

For isolation of total protein extracts, NSCs were collected and lysed using an ice-cold lysis buffer (10 mM Tris-HCl, pH 7.6, 5 mM MgCl_2_, 1.5 mM potassium acetate, 1% Nonidet P-40, 2 mM DTT, and protease inhibitors) for 30 min in ice. Samples were then sonicated for 30 s in ultrasounds, the lysate was centrifuged at 10,000 g for 10 min at 4°C, and the supernatant recovered. For isolation of mitochondrial protein extracts, cells were lysed upon incubation in ice for 20 min with an isolation buffer (20 mM HEPES, 10 mM KCl, 1.5 mM MgCl_2_⋅6H_2_O, 1 mM Na_2_EDTA, 1 mM EGTA, 250 mM Sucrose), supplemented with 1 mM dithiothreitol (DTT) and Halt Protease and Phosphatase Inhibitor Cocktail (Thermo Fisher Scientific, Inc.) and, then, disrupted by 40 strokes in a Dounce homogenizer. The homogenates were centrifuged at 2,500 g for 10 min at 4°C, then, the supernatant was saved and a second centrifugation was necessary to increase the product yield, so the pellet obtained from the homogenates was resuspended again in a minimum volume of isolation buffer. The total homogenate recovered was, then, centrifuged at 12,000 g for 30 min at 4°C to remove unbroken cells and nuclei. Finally, the mitochondrial fraction was obtained, the respective pellet resuspended in the isolation buffer and frozen at –80°C. The supernatant was removed and filtered through 0.2 μm and, then, 0.1 μm Ultrafree MC filters (Merck Millipore Corp., Germany) by centrifugation at 12,000 g for 20 min at 4°C to obtain cytosolic proteins. For nuclear extracts, cells were lysed with hypotonic buffer (10 mM Tris-HCl, pH 7.6, 5 mM MgCl_2_, 1.5 mM potassium acetate, 2 mM DTT, and protease inhibitors), homogenized with 20 strokes in a loose fitting Dounce homogenizer, and centrifuged twice at 500 g for 10 min at 4°C. Cytosolic proteins were recovered in the supernatant and centrifuged at 3,160 g for 10 min at 4°C saving the supernatant again, while the nuclear pellet was washed in a buffer composed of 10 mM Tris-HCl, pH 7.6, 5 mM MgCl_2_, 0.25 M sucrose, 0.5% Triton X-100, and protease inhibitors through centrifugation at 500 g for 5 min at 4°C. Then, the nuclear pellet was resuspended and sonicated for four cycles of 10 s in buffer composed by 10 mM Tris-HCl, pH 7.6, 0.25 M sucrose and protease inhibitors. Finally, the suspension was centrifuged through 0.88 M sucrose at 2,000 g for 20 min at 4°C, and nuclear proteins were recovered in the supernatant. Protein content was measured by the Bradford protein assay kit (Bio-Rad Laboratories, United States), according to the manufacturer’s specifications, using bovine serum albumin (BSA) as standard.

### Histone Purification

For histone purification cells were lysed with hypotonic lysis buffer (10 mM Tris-HCl, pH 8, 1 mM KCl, 1.5 mM MgCl_2_, 1 mM DTT and protease inhibitors) for 30 min at 4°C. Intact nuclei were recovered by centrifuging at 10,000 g for 10 min at 4°C, and the supernatant discarded. Nuclei were resuspended in 0.4 N H_2_SO_4_ and incubated on a rotator at 4°C overnight. Nuclear debris were removed by centrifuging at 16,000 g for 10 min at 4°C, and the supernatant containing histones was transferred into a fresh tube. Trichloroacetic acid (100%) was added drop by drop to histone solution, mixed and incubated on ice for 30 min. Histones were recovered after centrifugation at 16,000 g for 10 min at 4°C. The histone pellet was washed twice with ice-cold acetone and air-dried for 20 min at room temperature. Histones were finally dissolved in 100 μL of Milli-Q water and transferred into a new tube. Histone protein content was measured by the Bradford protein assay kit (Bio-Rad Laboratories), according to the manufacturer’s specifications, using BSA as standard. Ponceau S (P7170; Sigma-Aldrich Corp.) was used to reversible stain total protein bands and as a loading control of histone extracts, since the total H3 content was also increased in TUDCA-treated NSCs.

### Immunoblotting

Protein levels GAPDH, acetyl-H3, histone 3, Hsp70, lamin B1, LCAD, PDHE1-α and SREBP-1 and VDAC were determined by western blot analysis. Briefly, 20 μg of protein from mitochondrial, nuclear, and histone protein extracts and 40 μg of cytoplasm protein fraction and total protein extracts were separated on a 12% sodium dodecyl sulfate-polyacrylamide electrophoresis gel and then subjected to immunoblotting using primary antibodies reactive to the different proteins of interest (Table 1). Blots were subsequently incubated with secondary antibodies conjugated with anti-mouse or anti-rabbit immunoglobulin G (IgG), and with horseradish peroxidase (Bio-Rad Laboratories) for 2 h at room temperature. Finally, membranes were processed for protein detection using Immobilon^TM^ Western Chemiluminescent HRP Substrate (Millipore Corp.) or Super Signal^TM^ West Femto substrate (Thermo Fisher Scientific, Inc.) in a ChemiDoc^TM^ MP System (Bio-Rad Laboratories). GAPDH, was used as loading control of total extracts. Ponceau S was used to reversible stain total protein bands. Since the levels of VDAC mitochondrial protein marker and H3 nuclear protein marker are likely to be altered in TUDCA-treated NSCs, Ponceau S was also used as a loading control of mitochondrial and nuclear extracts. Lamin B1, VDAC and GAPDH were used to confirm the purity of the cytosolic, nuclear and mitochondrial fractionation.

**TABLE 1:** Primary antibodies used for Western blot.

**Antigen**	**Host**	**Clonality**	**Company**	**Catalog number**	**Dilution**
GAPDH	Mouse	Monoclonal	Santa Cruz Biotec.	sc-32233	1:2500
Acetyl-H3	Rabbit	Polyclonal	Abcam plc	ab47915	1:1000
Total H3	Rabbit	Polyclonal	Millipore Corp.	06-755	1:500
Hsp70	Mouse	Monoclonal	ReD SYSTEMS	242707	1:10000
Lamin B1	Rabbit	Polyclonal	Abcam plc	ab16048	1:10000
LCAD	Rabbit	Monoclonal	Abcam plc	ab196655	1:10000
PDHE1-α	Mouse	Monoclonal	Abcam plc	ab110330	1:1250
SREBP-1	Mouse	Monoclonal	Abcam plc	ab3259	1:400
VDAC	Rabbit	Polyclonal	Cell Signaling Tech.	4866	1:1000

### Immunocytochemistry

For visualization of PDHE1-α cellular distribution, cells were incubated with 0.5 μM MitoTracker^®^ Red CMXRos (M-7512; Molecular Probes, Life Technologies Corp.), which preferentially accumulates in mitochondria, for 30 min at 37°C before cell harvesting. Cells were washed twice, fixed with 4% PFA in PBS, washed three times and then blocked for 1 h at room temperature in PBS, containing 0.1% Triton-X-100, 1% FBS, and 10% normal donkey serum (Jackson ImmunoResearch Laboratories, Inc., United States). Cells were incubated with a primary mouse monoclonal antibody reactive to PDHE1-α (ab110330, Abcam^®^) at a dilution of 1:200, overnight at 4°C. After three washes, the secondary DyLight 488-conjugated anti-mouse (211-482-171; Jackson ImmunoResearch) diluted at 1:200 was added to cells for 2 h at room temperature. Mouse NSC nuclei were stained with Hoechst 33258 (861405; Sigma-Aldrich Corp.) at 5 μg/mL in PBS, for 3 min at RT and, then, washed three times. Samples were mounted using Mowiol^®^ 4-88 (81381; Sigma-Aldrich Corp.) PDHE1-α cellular distribution in NSCs was evaluated using fluorescence microscopy assessments performed with a Zeiss AX10 microscope (Carl Zeiss Corp.), equipped with a 63x/1.4 oil plan-apochromat objective and an AxioCam HRm camera (Carl Zeiss Corp.). Images were processed using ImageJ.

### Immunohistochemistry

Brains were postfixed for 1 day in 4% PFA and then cryopreserved in 30% sucrose. For immunohistochemistry of NSC markers, brains were coronally processed in 30-μm-thick cryostat sections that were collected in a series of 10 slides. Each series contained an anterior-posterior reconstruction of four brain sections separated 300 μm between them. Slides were consecutively washed with 0.1 M phosphate buffer (PB) (0.2 M Na_2_HPO_4_; 0.2 M NaH_2_PO_4_; purified water; pH adjust between 7.2 to 7.4 with NaOH). Antigen retrieval was performed by 20 min incubation in 2 N HCl solution at 37°C followed by 10 min incubation in borate solution (pH 8.5). Slides were rinsed in PB solution and blocked for 1 h at RT in block solution (PB + 10% FBS + 0.2% Triton x-100 [10%]). For LCAD detection, slides were incubated overnight with the primary mouse monoclonal antibody anti-LCAD diluted in block solution (1:200) (17526-1-AP, Proteintech). Secondary antibody donkey anti-rabbit Alexa Fluor 568 diluted in block solution (1:400) (A10042; Life Technologies Ltd.) was incubated for 2 h. For DCX detection, slides were incubated overnight with the primary goat polyclonal antibody diluted in block solution (1:200) (sc-8066; Santa Cruz Biotechnology). Subsequently, secondary antibody biotinylated anti-goat IgG diluted in PB (1:200) (BA-9500; Vector Laboratories) was incubated for 30 min. Immunoreactivity was then detected with Cy^TM^2-conjugated streptavidin-conjugated anti-goat diluted in PB (1:200; Jackson ImmunoResearch Laboratories Inc.) and incubated for 30 min. For negative controls, the primary antibody was omitted during the staining. Nuclei were counterstained with Hoechst 33342 (1:1000 in water) (Invitrogen Corp.). The images were acquired using Leica DMi8 confocal fluorescence microscope and processed using ImageJ.

### Oil-Red-O Staining

The intracellular accumulation of lipids in NSCs was evaluated by Oil-Red-O (ORO)/isopropanol method (Fukumoto and Fujimoto, 2002). Free FAs mixture (2:1, oleate: BSA-palmitate) in a concentration of 500 μM was added to NSCs for positive control, also 500 μM of BSA for endogenous control. The work solution of ORO (O-0625; Sigma-Aldrich Corp.) was prepared, ORO was dissolved in ≥99.8% isopropanol (109634; Merck Millipore Corp) and left overnight at room temperature. The solution was filtered with cellulose acetate membrane syringe filter, pore size 0.45 μm (1520014; Frilabo). Three parts of ORO solution were added to 2 parts of bidistilled H_2_O. The solution was then filtered, left to stand for 30 min, and filtered again before use. Before staining, the dishes were then gently rinsed with PBS. One milliliter of 4% (w/v) PFA was added to each dish for 20 min to fix the cells at room temperature. Each dish was then rinsed twice with PBS and once with 60% (v/v) of isopropanol. ORO stain was applied for 5 min. The dishes were rinsed with PBS, followed by the addition of Mayer’s hematoxylin solution (MHS-32; Sigma-Aldrich Corp.) for 2 min. The dishes were then rinsed with PBS, air dried and mounted under a glass coverslip with 30 μL PBS/glycerol (3:1, v/v). The staining effectiveness was evaluated with Zeiss Axioskop 50 microscope (Carl Zeiss Corp., Germany) inverted research microscope equipped with Axiocam 105 color (Carl Zeiss Corp.). Images (400x) were captured, enhanced and quantified using ImageJ.

### Liquid Chromatography Mass Spectrometry-Based Proteomics

To investigate the impact of TUDCA on the mitochondrial proteome in self-renewing and differentiating mouse NSCs, we performed liquid chromatography coupled with mass spectrometry (LC-MS). Briefly, NSC line NS-TGFP was expanded and induced to differentiate for 24 h with or without TUDCA. Mitochondrial protein enriched extracts were obtained by using Qproteome Mitochondria isolation kit (Qiagen) and resuspended in 6 M urea and 50 mM ammoniumbicarbonate. Protein tryptic digestion was performed according to (Llombart et al., 1996). Shortly, disulfide bonds were reduced by DTT and cysteine residues alkylated with iodoacetamide. Samples were digested with trypsin (Promega) and the reaction was stopped by addition of 0.5% formic acid. A total of 2 μg of each digest was first separated by a nano-HPLC system (Proxeon, Denmark) and then the peptide mass spectra acquired using a Maxis Impact Q-TOF spectrometer (Bruker, Bremen, Germany). The peptides were first concentrated on a 100 μm ID 2 cm nano-trapping column (Proxeon) and then loaded onto a 75 μm ID, 25 cm Acclaim PepMap nano-separation column (Thermo). The chromatography run using a 0.1% formic acid – acetonitrile gradient (2–30% in 120 min at a flow rate of 300 nL/min). The column was coupled to the mass spectrometer inlet through a Captive Spray ionization source (Bruker). MS acquisition was set to cycles of MS, each followed by 3 cycles of MS/MS, with an intensity threshold for fragmentation of 2,000 counts, and using a dynamic exclusion time of 2 min, with an automated precursor re-selection when a 3-fold increase in intensity was observed. Spectra were acquired on the range of 150–2,200 Da. LC-MS/MS data were pre-processed using the Data Analysis 4.2 software (Bruker). Data normalization, database searching, and protein identification were performed using MaxQuant software (v1.5.2.8) and protein quantification was determinate by MaxLFQ algorithm. Searches on a mouse database retrieved from Uniprot/SwissProt or NCBI database (downloaded on 2016, containing 166,073 reviewed sequences) included trypsin as digesting enzyme with a maximum of 2 missed cleavages; cysteine carbamidomethylation set as fixed modification and methionine oxidation as variable modification. The peptide mass tolerances of the first search and main search (recalibrated) were <0.07 and 0.006 Da, respectively. The minimum peptide length was seven amino acids, and the maximum peptide mass was 4,600 Da. Both peptides and proteins were filtered with a maximum false discovery rate (FDR) of 0.01. The match between runs feature with a matching window of 0.7 min and an alignment window of 20 min, was activated. Label-free quantitation (LFQ) calculations were performed separately in each parameter group containing similar cell loadings. All peptides were selected for protein quantification. Other unmentioned parameters were the MaxQuant default settings. Potential contaminants and reverse sequences were filtered out. The extracted data were further processed with Metaboanalyst. The mass spectrometry proteomics data have been deposited to the ProteomeXchange Consortium via the PRIDE partner repository with the data set identifier PXD017979. All MS analysis procedures were performed at the Laboratori de Proteoòmica, Vall d’Hebron Institut d’Oncologia (VHIO), Institut de Recerca Hospital Univ. Vall d’Hebron, Barcelona, Spain. A BLASTp search was performed through the BLAST2GO software (BioBam). This search retrieved the gene ontology (GO) annotations of the identified proteins by using GO categories (cellular location, molecular function and biological process) of the best hit derived from the BLASTp results (BLASTp minimal expectation value set to <1 × 10^–3^). Assignment of experimental condition-specific pathways and biological processes were performed inSTRING (Search tool for the retrieval of interacting genes/proteins) database. False Discovery Rate of the group of proteins belonging to UniProt Keywords and Biological Process GO was <0.001.

### Gas Chromatography–Mass Spectrometry Analysis of Fatty Acids

The qualitative and quantitative study of individual FA was performed using Gas Chromatography-Mass Spectrometry (GC-MS) (Shimadzu QP2010 Plus) according to Costa et al. (1998). Analyses were performed either in scanning or single ion monitoring (SIM) acquisition modes, where the obtained retention parameters and mass spectra were interpreted using an in-house MS library. Internal standard (IS) method was used for quantification (Sigma Chemical Co., United States). For the analysis of metabolites including the most important saturated, unsaturated and hydroxylated short-, medium- and long-chain FA up to C18, cells were collected by scraping in ice-cold 80% (v/v) of methanol (106009; Merck Millipore Corp). The respective homogenate was washed three times and centrifuged at 12,000 g for 10 min at 4°C. The supernatant was evaporated to dryness under a nitrogen stream and derivatized with N-methyl-N-(tert-butyldimethylsilyl)-trifluoroacetamide (MTBSTFA): methyl-bis(trifluoroacetamide) (MBTFA): Pyridine (4:5:1) (Pierce-Thermo Scientific, United States) at 60°C for 1 h. Samples were injected with an automatic injector (AOC 20i), using the split injection mode (1/50 ratio). Acquisition in SIM mode was based on specific masses (*m*/*z*) selected as function of respective FA fragmentation spectrum. The peak area ratios (analyte/IS) were normalized to total protein content (mg). Data are expressed as fold-change relative to untreated cells (control). Total protein extracts were measured by the Pierce^TM^ BCA Protein Assay Kit (Thermo Fisher Scientific, Inc.), according to the manufacturer’s specifications, using BSA as standard.

### Transfection Assays

For the RNA interference of LCAD, NS-TGFP were transfected in differentiation induced medium with 60 nM of small interfering RNA (siRNA) specific for LCAD (Acadl Silencer Pre-designed siRNA cat# AM16708 ID162072, Ambion) or the respective negative control (Silencer^®^ Select Negative Control #1 siRNA cat#4390844, Ambion) with Lipofectamine^TM^ 3,000 (Invitrogen Corp.), 24 h after platting. At the end of treatments, cells were detached with accutase and collected for analysis of NSC fate. To assess silencing efficiency, total levels of LCAD were determined by Western blot (Supplementary Figure S1A).

### Total RNA Extraction

Total RNA was extracted using the RIBOZOL^TM^ reagent (AMRESCO, LLC, Solon, OH, United States) according to manufacturer’s instructions. Each sample was homogenized on 0.5 mL RIBOZOL^TM^ reagent. After mixing with 0.1 mL chloroform, each sample was centrifuged at 12,000 g for 15 min at 4°C and the aqueous phase collected. Total RNA was precipitated by incubation with 0.25 mL isopropyl alcohol at −20°C for 1 h. Samples were centrifuged at 12,000 g for 10 min at 4°C and the RNA pellet was washed with 75% ethanol and centrifuged at 7,500 g for 5 min at 4°C. RNA pellets were air dried and resuspended in 40 μL RNase-free water. The purity of RNA was checked and DNA contaminations were eliminated with DNase I recombinant (04716728001; Roche Applied Science, Mannheim, Germany) following manufacturer’s instructions.

### Quantitative RT-PCR (qRT-PCR)

cDNA synthesis was performed using the NZY Reverse Transcriptase (NZYTech, Lisbon, Portugal) according to manufacturer’s instructions. Real-time RT-PCR was performed using SensiFastTM SYBR^®^ Hi-ROX Kit (Bioline USA Inc., Taunton, MA, United States) in the Applied Biosystems 7300 System (Thermo Fisher Scientific Inc.). Primer sequences can be found in Table 2. Relative gene expression was calculated based on the standard curve and normalized to the level of hypoxanthine phosphoribosyltransferase (*HPRT*) housekeeping gene and expressed as fold change from controls.

**TABLE 2:** List of primers used for qPCR.

	**Sequence (5′–3′)**
*acc1*	5′-GGA CAC CAG TTT TGC ATT CA-3′ (fwd)
	5′-AGT TTG GGA GGA CAT CGA AA-3′ (rev)
*nestin*	5′– CTC AGA TCC TGG AAG GTG GG-3′ (fwd)
	5′-GCA GAG TCC TGT ATG TAG CCA-3′ (rev)
*ki67*	5′-CCT TTG CTG TCC CCG AAG A-3′ (fwd)
	5′-GGC TTC TCA TCT GTT GCT TCC T-3′ (rev)
*map-2*	5′-GTT CAG GCC CAC TCT CCT TC-3′ (fwd)
	5′-CTT GCT GCT GTG GTT TTC CG-3′ (rev)
*hprt*	5′-GGT GAA AAG GAC CTC TCG AAG TG -3′ (fwd)
	5′-ATA GTC AAG GGC ATA TCC AAC AAC A-3′ (rev)

### Densitometry and Statistical Analysis

In proteomics analyses, significance threshold was set to *p* < 0.05, minimum MASCOT ions score of 20. Protein identifications with only one peptide with 95% confidence were performed using two additional quality criteria: sequence coverage of ≥10% and a deviation of predicted mass, RMS90 of ≤50 ppm. The relative intensities of protein bands in immunoblotting were analyzed using the Image Lab Version 5.0 densitometric analysis program (Bio-Rad Laboratories). Results were compared using an unpaired Student’s *t*-test. Values of *p* < 0.05 were considered statistically significant. Statistical analysis was performed with GraphPad Prism 6.1 software (GraphPad Software, Inc., United States).

## Results

### TUDCA Decreases LCAD Protein Levels in Early Differentiation of NSCs

We have shown that the endogenous bile acid TUDCA is a well-known mitochondrion protecting agent in early stages of neural differentiation (Xavier et al., 2015), while promoting the proliferation of NSCs, both *in vitro* and *in vivo*, to increase NSC pool (Xavier et al., 2014; Soares et al., 2018). However, these intriguing findings await further exploration to better understand how NSC fate is controlled by mitochondria.

**FIGURE 1:**
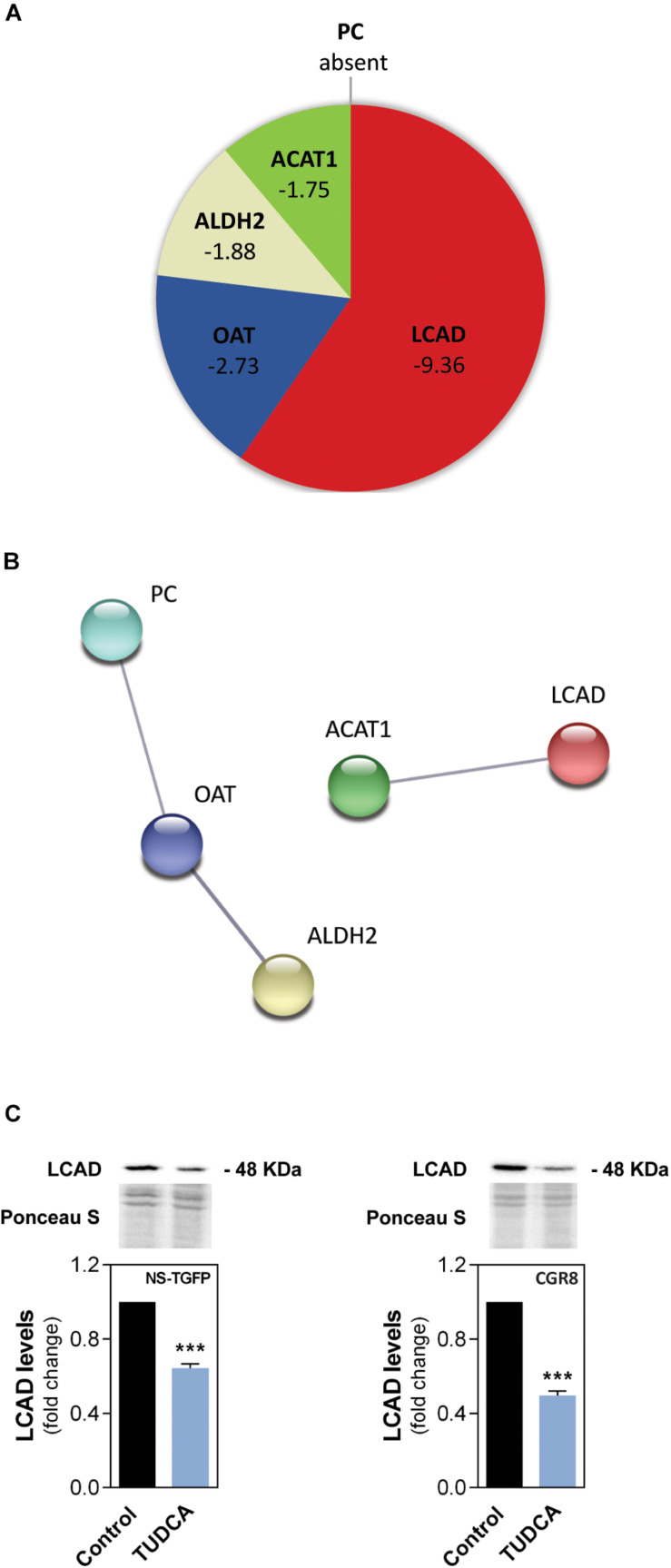
The proliferation-inducing agent TUDCA downregulates a FA oxidation-associated enzyme in NSCs. Early-differentiating mouse NSC lines NS-TGFP and CGR8 were incubated with TUDCA for 24 h. Mitochondrial protein purified extracts were isolated for liquid chromatography mass spectrometry-based proteomics (LC-MS) and/or Western blot analysis, as described in Materials and Methods. **(A)** Pie diagram of the proteomic data showing the mitochondrial proteins significantly regulated by TUDCA in NS-TGFP cell line, and respective variations, when compared with control. The proteins were identified as the long-chain acyl-CoA dehydrogenase (LCAD), the ornithine aminotransferase (OAT), the aldehyde dehydrogenase 2 (ALDH2), the acetyl-CoA acetyltransferase (ACAT1) and the pyruvate carboxylase (PC). Results are expressed as mean in fold-change, for at least three independent experiments, when compared with control (untreated cells). Significance threshold was set to *p* < 0.05 from control (untreated cells). **(B)** STRING analysis showing protein-protein network between the most regulated mitochondrial proteins by TUDCA in NSCs. Nodes represent proteins and lines connecting nodes indicate direct, or indirect interactions between proteins. Colorful nodes represent proteins that are downregulated or absent in TUDCA-treated NSCs. Functional protein network database version 10.0 (http://string-db.org, accessed in August 2019). **(C)** Representative immunoblots (*top*) of LCAD mitochondrial levels and respective densitometry analysis (*bottom*) in mitochondrial protein extracts from the NSC lines. LCAD levels were normalized to Ponceau S. Data are expressed as mean ± SEM fold-change for at least three independent experiments. ***p < 0.001 from control (untreated cells).

Therefore, we investigated the impact of TUDCA on the mitochondrial proteome in mouse NSCs by high-throughput proteomics analysis using liquid chromatography coupled with mass spectrometry (LC-MS). Proteomics data revealed possible novel targets downregulated by TUDCA in NSCs. Surprisingly, most of these mitochondrial proteins were significantly downregulated or even absent in the presence of TUDCA in NSCs (Figure 1A and Supplementary Table S1). We identified proteins involved in cell metabolism, such as aldehyde dehydrogenase 2 (ALDH2) responsible for converting acetaldehyde metabolized from ethanol to acetate, and the acetyl-CoA acetyltransferase (ACAT1, also known as β-thiolase) that catalyzes the condensation of two acetyl-CoA to acetoacetyl-CoA, as well as the reverse reaction, by breaking down acetoacetyl-CoA (Fukao et al., 1990). Also, the pyruvate carboxylase (PC), which forms oxaloacetate for anaplerotic purposes (Jitrapakdee et al., 2008) was absent in TUDCA-treated NSCs. Curiously, neurons lack PC (Danbolt, 2001; Hertz and Zielke, 2004) and TUDCA-induced PC ablation could represent part of the mechanism by which TUDCA primes NSCs toward neuronal fate (Xavier et al., 2014; Soares et al., 2018). The ornithine aminotransferase (OAT), which participates in amino acid metabolism was also shown to be downregulated by TUDCA. The main function of OAT is to control the production of signaling molecules, such as glutamate (Ginguay et al., 2017).

In relation with FA metabolism, TUDCA was shown to markedly downregulate the long-chain acyl-CoA dehydrogenase (LCAD). In fact, LCAD and ACAT1 can be involved in the β-oxidation of FAs. LCAD catalyzes a key initial step in mitochondrial FAO, acting on C8-C20 FAs (Kompare and Rizzo, 2008), while ACAT1, acts downstream to LCAD (Houten et al., 2016). Interestingly, STRING analysis suggested that all mitochondrial proteins downregulated by TUDCA are somehow involved in acetylation-related processes (UniProt Keywords GO number KW-0007) predicting functional links between proteins, such as between LCAD and ACAT1 (Figure 1B). Although LCAD and ACAT1 do not physically interact, the STRING analysis also revealed that they are co-expressed in the same manner, indicating a possible general inhibition of lipid catabolism by TUDCA in NSCs. In mice, LCAD plays an essential role in the degradation of polyunsaturated fatty acids (PUFAs) and of branched-chain FAs. Thus, this protein seems to play a more redundant role in the oxidation of long- and straight- chain saturated FAs (van Vlies et al., 2005; Chegary et al., 2009). To further understand the significance of the β-oxidation in NSC fate changes induced by this proliferation-inducing agent we validated the TUDCA-downregulated LCAD from proteomics data. The validation was performed by Western blot analysis in the same mouse NSC line (NS-TGFP) as well as in an additional one (CGR8). Our results showed that TUDCA significantly decreased LCAD protein levels by ∼40 and 50% in NS-TGFP and CGR8 cell lines, respectively, when compared to the respective controls (Figure 1C). Therefore, our results revealed that TUDCA markedly reduces LCAD in NSCs, an enzyme pivotal for β-oxidation of long-chain FAs.

### LCAD Downregulation Is Associated With Increased NSC Proliferation

How metabolism is reprogrammed during NSC fate remains largely unknown. On the other hand, neural differentiation is a multistep process that shapes and re-shapes NSCs by progressing through several typical stages. Thus, we evaluated the expression of LCAD throughout the early stages of NSC differentiation by immunoblotting analysis, and investigated the impact of LCAD silencing on NSC proliferative and differentiation potential by qRT-PCR.

**FIGURE 2:**
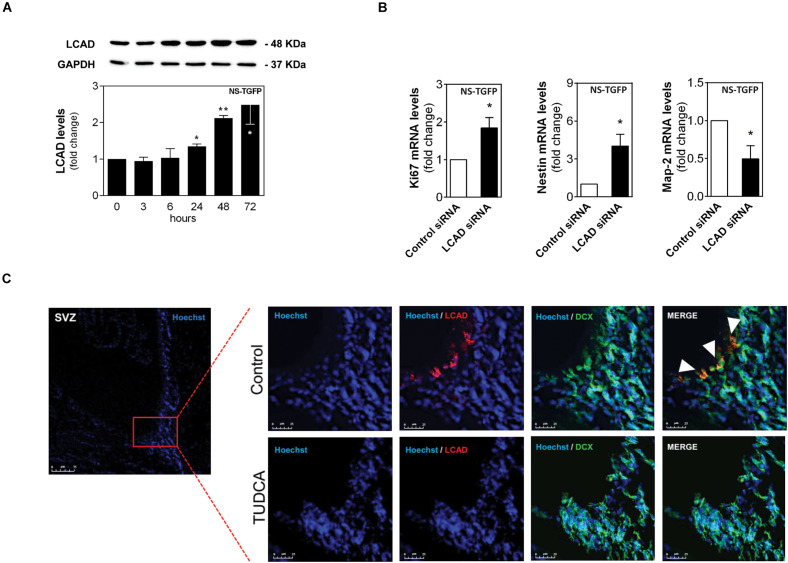
LCAD downregulation is associated with increase of NSC proliferation. The NSC line NS-TGFP was induced to differentiate for up to 72 h and total proteins were collected for immunoblotting analysis. NSCs were also transfected with either control or LCAD siRNAs, and collected for RT-PCR analysis to assess mRNA expression levels of proliferation, stemness and differentiation markers after 24 h. In addition, adult rats were treated with TUDCA or vehicle for 28 days using miniosmotic pumps, as described in Materials and Methods. Immunohistochemistry against LCAD (red) and NSC early differentiation marker DCX (green) was performed. **(A)** Representative immunoblots of total LCAD levels (top) and corresponding densitometry analysis (bottom) in NSCs throughout differentiation. Total LCAD levels were normalized to GAPDH. **(B)** mRNA expression levels of proliferation (ki67), stemness (Nestin) and differentiation (Map-2) markers in NSCs collected 24 h after siRNA transfection. Values were normalized to the internal standard HPRT. Data are expressed as mean ± SEM fold-change for at least three independent experiments. **p < 0.005 and *p < 0.05 from control (untreated cells). **(C)** Representative confocal images of LCAD- and DCX-positive cells in frontal sections of adult SVZ neurogenic niches in TUDCA-treated and untreated rats (*n* = 4). Nuclei were stained with Hoechst. Scale bars, 75 μm in SVZ image and 25 μm in representative image sections with or without TUDCA treatment.

Our results showed that LCAD protein levels gradually increased up to 2.5-fold (Figure 2A), after 3 days of NSC differentiation, when compared to undifferentiated cells. More importantly, we found that 50% of LCAD downregulation (Supplementary Figure S1A) almost doubled the proliferative marker ki67 of NSCs and significantly decreased neurogenesis progression. In fact, siRNA-induced silencing of LCAD in NSCs resulted in a marked increase of the undifferentiation marker, Nestin and a significant decrease of the neuronal marker Map-2 (Figure 2B). The transiently inhibition of LCAD expression in transfected NSCs was validated by Western blot after 24 h (Supplementary Figure S1A).

We then investigated whether LCAD expression was indeed decreased in the neurogenic niches of adult rat brains after TUDCA exposure (Figure 2C). Hence, extra brain slices collected from our previous study, revealing the proliferative effects of TUDCA *in vivo* (Soares et al., 2018), allowed the assessment of TUDCA effect on LCAD levels in the same animals through immunohistochemistry staining against LCAD in the subventricular zone (SVZ) neurogenic region. Notably, we found that after 28 days of TUDCA administration in adult rat brains using mini-osmotic pumps induced a significant decrease of this specific FAO-related enzyme in SVZ neurogenic regions, corroborating the negative effect of TUDCA on LCAD in cells. Thus, our results support the idea that TUDCA increases the NSC pool by repressing this specific enzyme suggesting decreased flux of long-chain FAs through mitochondrial β-oxidation.

### TUDCA Stimulates *de novo* Lipogenesis in NSCs

**FIGURE 3:**
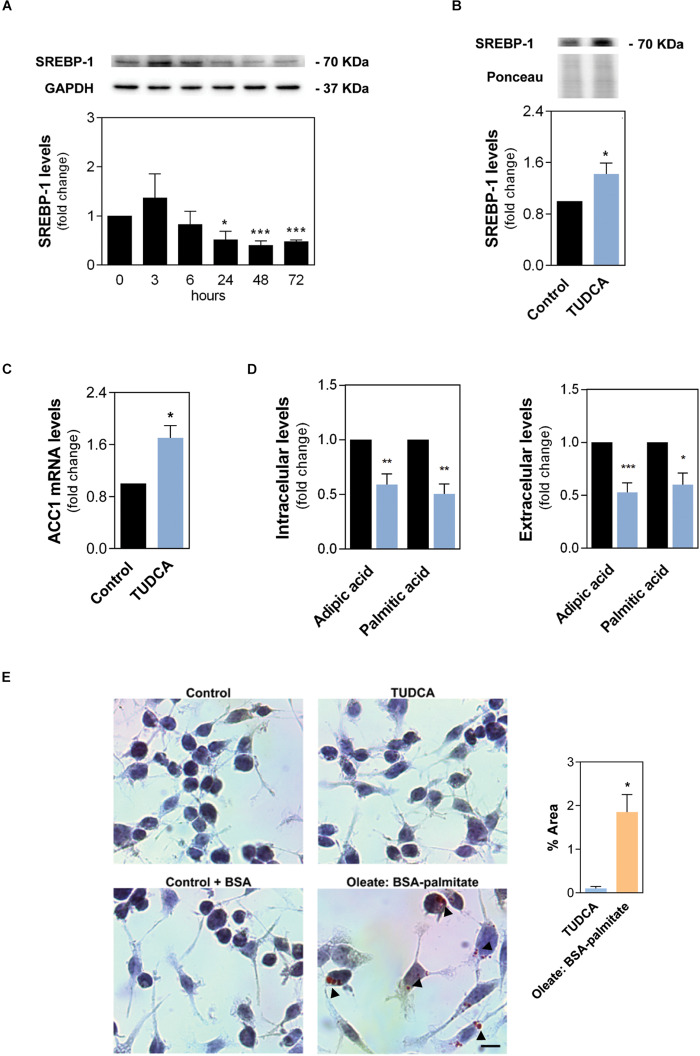
The proliferation-inducing agent TUDCA supports *de novo* lipogenesis in NSCs. The NSC line NS-TGFP was induced to differentiate for up to 72 h and total proteins were collected for immunoblotting analysis. Total and nuclear protein extracts were isolated for Western blot analysis. Early-differentiating mouse NSCs were also incubated with TUDCA for 24 h and collected for Western blot, RT-PCR, GC-MS analysis and Oil-Red-O staining, as described in Materials and Methods. **(A)** Representative immunoblots of total SREBP-1 levels (top) and corresponding densitometry analysis (bottom) in NSCs throughout differentiation. Total SREBP-1 levels were normalized to GAPDH. Data are expressed as mean ± SEM fold-change for at least three independent experiments. *p < 0.05 and ***p < 0.001 from undifferentiated cells. **(B)** Representative immunoblots of SREBP-1 levels (top) and corresponding densitometry analysis (bottom) in nuclear protein extracts. Nuclear SREBP-1 levels were normalized to Ponceau S. **(C)** mRNA expression levels of ACC1 lipogenesis marker in NSCs collected 24 h after TUDCA exposure. Values were normalized to the internal standard HPRT. **(D)** Cellular amount of palmitic and adipic acid in differentiating NSCs detected by gas chromatography coupled with mass spectrometry (GC-MS) analysis. The peak-area ratios (sample/internal standard) were normalized to total protein content (mg). Data are expressed as mean ± SEM fold-change for at least three independent experiments. *p < 0.05, **p < 0.005, ***p < 0.001 from control (untreated cells). **(E)** Representative images of the lipid droplets in red (indicated by arrows) in NSCs and respective quantification. Free FAs mixture (oleate: BSA-palmitate) and BSA were added to NSCs for positive and endogenous control, respectively. Nuclei were stained with hematoxylin. Scale bar, 25 μm. Data are expressed as mean ± SEM fold-change for at least three independent experiments. *p < 0.05 from TUDCA-treated cells.

To further clarify how TUDCA modulates the balance between FA oxidation and respective biosynthesis in NSCs, we investigated protein levels of the sterol regulatory element-binding protein 1 (SREBP-1) in NSCs, since this protein is a key transcription factor involved in lipogenesis (Eberlé et al., 2004). Curiously, at odds with results previously obtained for LCAD, SREBP-1 protein levels decreased by ∼50% (Figure 3A) after 3 days of differentiation, when compared to undifferentiated cells. More importantly, in the same previous conditions, TUDCA significantly increased the levels of nuclear SREBP-1 constitutively active form, when compared to control (Figure 3B), suggesting that proliferating NSCs drive cell metabolism to favor lipogenesis rather than lipid oxidation. The TUDCA impact in lipogenesis was corroborated by qRT-PCR experiments in NSCs treated and untreated with TUDCA. Interestingly, our results indicated that TUDCA not only increases SREBP, but also significantly enhances mRNA levels of other lipogenesis-associated genes, such as the acetyl-CoA carboxylase 1 (ACC1) (Figure 3C), which in turn connects central energy metabolism to lipid biosynthesis and codes for a protein that is rate-limiting for the *de novo* synthesis of lipids.

To further dissect the metabolic alterations undergoing proliferation, we then compared the intracellular and extracellular FA levels of TUDCA-treated and untreated NSCs. Interestingly, after 24 h of TUDCA treatment, we observed an ∼50% reduction in total levels of dicarboxylic saturated medium chain FA (C6:0; adipic acid) and hexadecanoic acid (C16:0, palmitic acid; PA) (Figure 3D), while no significant differences in other relevant saturated or unsaturated long-chain fatty acids (up to C18:0) were observed (data not shown). On extracellular media, milder significant decreases were also found in saturated octanoic (C8:0), decanoic (C10:0), dodecanoic (C12:0) (data not shown). Of note, palmitate serves as building lipid blocks and can be elongated and unsaturated to form more complex lipids who may have critical cell signaling functions (Bieberich, 2012; Knobloch, 2016). It is important to note that, although the marked decrease in palmitic levels may indicate a reduction in lipogenesis rates, it might also indicate a high consumption of this metabolite to assure lipid synthesis progress. Nevertheless, to understand TUDCA impact on NSC lipid content we evaluated the intracellular lipid accumulation in NSCs treated and untreated with TUDCA by staining NSCs with Oil-Red-O (ORO). In fact, accumulation of oleic acid was recently shown to decrease NSC proliferation in an Alzheimer’s disease mouse model (Hamilton et al., 2015). Interestingly, NSCs did not show any sign of lipid accumulation after 24 h of TUDCA treatment, having a similar appearance compared to control. In contrast, NSCs treated with long-chain FAs, such as oleate: BSA-palmitate, used as a positive control, showed intracellular major lipid droplets around the nucleus whereas the relative control was clear of lipid accumulation (Figure 3E). These results show that TUDCA induces a major upstream regulator of lipogenesis, without increasing lipid droplets, which can be important to enable generation of lipid membranes and stimulate the proliferation activity of NSCs.

### TUDCA Increases PDHE1-α and Potentiates a Novel Mechanism for Its Mitochondria-Nucleus Translocation in NSCs

Next, we attempted to unravel the possible impact of TUDCA in cellular energy production of NSCs through glucose metabolism and oxidation. In fact, we hypothesized that a decreased β-oxidation of long-chain FAs induced by this bile acid might ignite a metabolic shift in these cells, as a strategy to ensure energy production.

**FIGURE 4:**
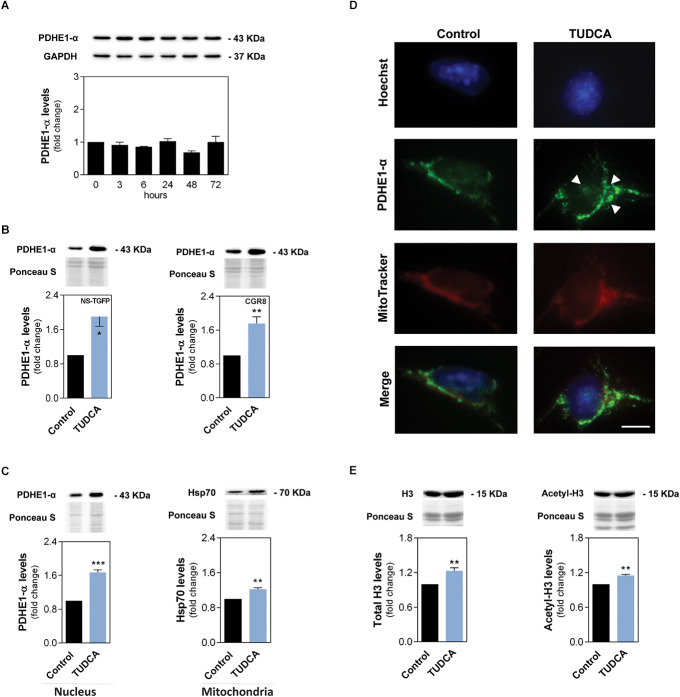
TUDCA increases mitochondrial and nuclear levels of PDHE1-α in NSCs while supporting acetylation of increased H3. The NSC line NS-TGFP was induced to differentiate for up to 72 h and the total protein collected for immunoblotting analysis. Early-differentiating NSCs were also incubated with TUDCA for 24 h and collected for Western blot and immunocytochemistry, as described in Materials and Methods. **(A)** Representative immunoblots of total PDHE-1α levels (top) and corresponding densitometry analysis (bottom) in NSCs throughout differentiation. Total PDHE-1α levels were normalized to GAPDH. Data are expressed as mean ± SEM fold-change for at least three independent experiments. **(B)** Representative immunoblots of PDHE1-α levels (top) and corresponding densitometry analysis (bottom) in mitochondrial protein extracts of NS-TGFP and CGR8 cell lines. **(C)** Representative immunoblots of PDHE1-α (right) and Hsp70 (left) levels (top) and corresponding densitometry analysis (bottom) in nuclear and mitochondrial protein extracts, respectively. **(D)** Representative images of immunofluorescence detection of cells labeled with an anti-PDHE1-α antibody (green). Nuclei were stained with Hoechst 33258 (blue) and mitochondria with Mitotracker (red). Scale bar, 20 μm. **(E)** Representative immunoblots of total H3 (left) and acetyl-H3 (right) levels (top) and corresponding densitometry analysis (bottom) in purified histone extracts from NS-TGFP cells. Specific mitochondrial and histone protein levels were normalized to Ponceau S. Data are expressed as mean ± SEM fold-change for at least three independent experiments. *p < 0.05, **p < 0.01, ***p < 0.001 from control (untreated cells).

The pyruvate dehydrogenase complex (PDC) provides the primary link between glycolysis and the tricarboxylic acid cycle (TCA) cycle by catalyzing the irreversible conversion of pyruvate into acetyl-CoA (Varum et al., 2011). The pyruvate dehydrogenase E1-α (PDHE1-α) contains the E1 active site from PDC, therefore playing a key role in its function. Thus, we investigated possible changes of PDHE1-α subunit during early stage NSC differentiation. Our results showed that, although the total levels of PDHE1-α did not show any substantial differences throughout differentiation induction (Figure 4A), TUDCA is capable of significantly increasing mitochondrial levels of PDHE1-α in differentiating NSCs. In NS-TGFP and CGR8 cell lines, PDHE1-α levels increased almost 2-fold, when compared to controls (Figure 4B). Upregulation of PDHE1-α by TUDCA appears to indicate that the conversion of pyruvate into acetyl-CoA is favored in mitochondrial matrix.

Nuclear PDHE1-α exists in human normal and cancer cells as a critical step to generation of acetyl-CoA and histone acetylation in mitotic phase (Sutendra et al., 2014). Thus, we decided to also explore this possibility in our context and, surprisingly, our results showed that PDHE1-α was indeed present in the nucleus of NSCs and further accumulated after TUDCA treatment. In fact, TUDCA markedly induced nuclear levels of PDHE1-α, increasing ∼70% when compared to control (Figure 4C).

The cellular distribution of PDHE1-α in NSCs was also evaluated by immunocytochemistry, colocalizing with both mitochondria organelles and nuclei. In fact, PDHE1-α was detectable in the nucleus in both untreated and TUDCA-treated NSCs. Corroborating our previous results, TUDCA-induced upregulation of PDHE1-α was observed both in mitochondria and in the nucleus, when compared to control (Figure 4D). The purity of mitochondrial and nuclear fractionation was validated by Western blot (Supplementary Figure S1B).

The mechanism of PDC mitochondrial-nuclear translocation has been shown to be dependent on the chaperone Hsp70, which facilitates its nuclear import through association with E1 and E2, two of the three enzymes of PDC. In fact, induction of Hsp70 is cell cycle-dependent, with its highest expression also observed during S phase (Sutendra et al., 2014; Tang, 2015). In agreement, immunoblotting analysis demonstrated that Hsp70 is expressed in mitochondria and its levels are increased in NSCs treated with TUDCA (Figure 4C).

Acetyl-CoA generated by PDC in the nucleus is apparently important for histone acetylation, as required for S phase entry and cell cycle progression (Tang, 2015). Moreover, TUDCA enhances NSC proliferation by modulating cell cycle progression, decreasing cells in G0-G1 phase and markedly increasing S/G2-M phases 24 h after the induction of NSC differentiation (Xavier et al., 2014). Thus, we finally assessed the acetylation levels of histone 3 (H3) in NSCs. Our results demonstrated that H3 total levels slightly increased in the presence of TUDCA. In addition, the levels of histone 3 acetylation were also similarly increased by TUDCA (Figure 4E). This indicates that TUDCA-induced H3 expression is accompanied by an increase in H3 acetylation levels. However, when acetyl-H3 levels were normalized to total levels of H3 (data not shown), we did not detect any significant difference in acetyl-H3 levels by TUDCA treatment, indicating that the increase in the acetylation process is, indeed, dependent on H3 total expression levels. The purity of histone extracts was carefully certified (Supplementary Figure S1C). These results strongly support the idea that TUDCA induces glucose oxidation (via pyruvate decarboxylation) and subsequent increases nuclear PDC levels for histone acetylation in proliferating NSC (Graphical Abstract).

## Discussion

Adult neurogenesis contributes to maintain physiological homeostasis and regeneration of damaged tissue in the brain (Ma et al., 2009). The decrease in the NSC pool is a primary factor for decline of both neurogenesis and its paracrine activity, throughout life (Kuhn et al., 1996). Thus, a better understanding of the mechanisms that regulate adult NSC behavior proves to be pertinent for the discovery of novel efficient strategies that enhance brain regeneration. The present work shows that the endogenous neuroprotective bile acid, TUDCA, a NSC proliferation-inducing agent, drives a metabolic remodeling involving pyruvate dehydrogenase nuclear translocation (Sutendra et al., 2014), previously speculated to also occur in stem cells (Lisowski et al., 2018).

Some of the molecular events involved in NSC differentiation are already well described; however, the bioenergetic demands and how strictly the energy metabolism is ruled to ensure adult NSC life-long activity is still unclear. TUDCA has been described as an antiapoptotic and antioxidant mitochondrial agent in NSCs and in mature neurons (Rodrigues et al., 2000; Xavier et al., 2014). Furthermore, this molecule was shown to enhance NSC proliferation and neuronal differentiation, both *in vitro* and *in vivo* (Xavier et al., 2014; Soares et al., 2018).

Here we deciphered the TUDCA mitochondrial proteomic signature in NSCs, demonstrating that this proliferation-inducing molecule also regulates pivotal metabolic-related proteins. Importantly, TUDCA was shown to markedly downregulate the levels of LCAD in early differentiating NSCs. Indeed, LCAD is highly expressed in the fetal brain, having a relative expression in the CA1 and CA4 areas and granular layer of human hippocampus, regions of adult neurogenesis and with proximal spatial relationship (Sohur et al., 2006; He et al., 2007). Moreover, it was recently demonstrated that LCAD activity is a source of FAO-driven H_2_O_2_ in mitochondria (Zhang et al., 2019). Accordingly, TUDCA as an antioxidant molecule is known to attenuate H_2_O_2_-induced reactive oxygen species generation (Xavier et al., 2014), which may indicate that TUDCA-mediated suppression on H_2_O_2_-induced oxidative stress perhaps happens through LCAD inhibition. Interestingly, hypoxia-inducible factor 1 (HIF-1) was recently shown to suppresses fatty acid β-oxidation in cancer cells through medium- and long-chain acyl-CoA dehydrogenases, hence sustaining proliferation and tumor progression. Again, this effect was revealed to be dependent on reactive oxygen species (ROS) levels controlling process (Zhang, 2015). In fact, it would be interesting, in the future, to clarify whether TUDCA controls LCAD expression by a HIF-1-mediated mechanism.

Our results also showed that the expression profile of LCAD increases during neural differentiation contrasting with the expression profile of the lipid biosynthesis-related SREBP-1 and ACC1. A possible link between FA oxidation and lipid synthesis could also be the cellular levels of palmitate. Indeed, although we detected an increase of LCAD at early stages of NSC differentiation, we did not have any evidence that palmitate levels increase at the same time-points. Since TUDCA exposure downregulates both LCAD and palmitic acid, we do not exclude this potential cross-link. However, in agreement with the increased LCAD levels throughout NSC differentiation, mitochondrial by-product ROS were already shown to be increased during neuronal differentiation in response to higher cellular energetic demands, also promoting transcription of neurogenic genes (Khacho et al., 2016). These results reflect the importance of β-oxidation as source of energy for differentiation. Mature neurons were shown to avoid extensive FAO and favor glucose oxidation in the brain (Schönfeld and Reiser, 2013). Although at the stage of cell differentiation, high rate of oxidative metabolism through oxidation of long-chain FAs no longer represents an alternative energy fuel, it might well support OXPHOS for energy production. In addition, recent findings support our view, indicating that manipulation of FAO is enough to instruct quiescent NSCs to enter the cell cycle and proliferate *in vitro* (Knobloch et al., 2017). In fact, an increase of LCAD expression in this context would likely result in quiescent NSC states or differentiation, negatively impacting the TUDCA-induced NSC proliferation. In addition, while some studies have shown that a FAO-dependent metabolic shift regulates adult NSC activity (Knobloch et al., 2017), others demonstrated that FAO promotes reprogramming by enhancing oxidative phosphorylation and inhibiting protein kinase C (Lin et al., 2018).

TUDCA, in turn, seems to favor NSC proliferation, by causing an inverse shift in the expression of SREBP-1 and LCAD confirming the role of TUDCA driving NSCs into a more proliferative state. Therefore, we conclude that TUDCA is able to trigger a metabolic shift from FA degradation toward lipid synthesis, which has already been associated with the proliferative activity of NSCs (Knobloch et al., 2012). Curiously, insulin may represent a clue for TUDCA induction of SREBP-1 expression, since TUDCA was shown to improve insulin signaling and sensitivity (Ozcan et al., 2006; Guo et al., 2015). Insulin *per se* is able to rapidly modulate SREBP-1c concentration in the nucleus by proteolytic cleavage activation (Eberlé et al., 2004). Further, insulin and the insulin-like growth factors (IGFs) support diverse essential roles in neurogenesis and proliferation of NSCs (Ziegler et al., 2015).

Interestingly, targeted MS-based analysis of FA suggests a significant TUDCA-associated decrease of palmitic acid. This long-chain FA is the major precursor for FA elongation. However, the decreased rate of mitochondrial long-chain FA oxidation due to LCAD repression may not necessarily predict accumulation of PA. In fact, in mice as in humans, the oxidative fate of palmitic acid may be held by other acyl-CoA dehydrogenases. Moreover, analysis of fibroblasts from LCAD^(–/–)^ mice has unequivocally revealed no significant changes in these types of metabolites, as compared with control (Chegary et al., 2009). Thus, our results suggest a highly dynamic control of NSC metabolic fluxes under LCAD downregulation, in which decreased levels of palmitic acid upon TUDCA treatment, might be explained by a rapid consumption of this metabolite, for FA elongation and *de novo* biosynthesis of FAs to enable generation of lipid membranes of high proliferating NSCs. Of note, SREBP-1 promotes the expression of two key enzymes, ACC whose mRNA levels we found to be upregulated in TUDCA presence, and fatty-acid synthase (FASN), for *de novo* FA synthesis. Interestingly, FASN is preferably distributed in major areas of neurogenic niches of the adult murine brain, where expression is high in proliferating NSCs and reduced in differentiated progeny (Knobloch et al., 2012). Importantly, we have shown that TUDCA while increasing lipid synthesis does not generate intracellular lipid accumulation in NSCs as levels of palmitic acid were known to reduce NSC proliferation and cause cell death (Park et al., 2011). Further, the accumulation of lipid droplets has been associated with cognitive disorders, including Alzheimer’s disease, and were already shown to directly interfere with NSC behavior, decreasing NSC proliferation (Hamilton et al., 2015).

Nevertheless, we were able to show that NSCs treated with the endogenous metabolic regulator TUDCA assure the energy supply by upregulating the expression of the major pyruvate dehydrogenase subunit, PDHE1-α. In fact, this molecule appears to interfere with the regulation of FA and glucose oxidation counterbalance, safeguarding the amount of substrate entering the TCA cycle through glucose oxidation. Notably, ACAT1 protein, whose mitochondrial levels are downregulated by TUDCA, was recently described to acetylate PDC and inactivate its function in cancer cells (Fan et al., 2016). Other explanation for TUDCA-induced upregulation of pyruvate dehydrogenase could be via regulation of its inhibitor pyruvate dehydrogenase kinase (PDK). Although we were not able to detect significant alterations in PDK levels in NSCs upon 24 h of TUDCA treatment (data not shown), we cannot exclude that TUDCA might reduce PDK levels at early time-points. On the other hand, PCG1-α, a major player of mitochondria biogenesis, is capable of increasing PDHE1-α protein content (Kiilerich et al., 2010). Thus, it is tempting to speculate that TUDCA-induced upregulation of PDHE1-α could be linked with the downregulation of ACAT1 protein expression and/or its already described effect on PCG1-α, a master regulator of mitochondrial biomass and activity (Fan et al., 2016; Soares et al., 2018).

Finally, we showed that TUDCA enhances a novel signaling pathway of mitochondrial-nuclear communication boosting nuclear PDHE1-α as a mitochondrial expatriate in NSCs. The TUDCA-induced NSC proliferation enhanced H3 supply that might be important for the S phase of cell cycle. Interestingly, histone acetylation may depend on different mechanisms for the generation of acetyl-CoA within the nucleus, such as the nuclear form of ATP citrate lyase using citrate (Wellen et al., 2009). In agreement, TUDCA was also found to up-regulate ATP citrate lyase transcript in primary rat hepatocytes (Castro et al., 2005). Indeed, nuclear PDC ability to generate acetyl-CoA may become important when the citrate pool is shifted toward lipid synthesis (Tang, 2015). Curiously, it was also recently demonstrated that PDC might directly contribute to the production of acetate, providing support for acetyl-CoA pools in all cellular compartments (Liu et al., 2018). Acetyl-CoA, in turn, facilitates lipid synthesis and histone acetylation processes (Shi and Tu, 2015) being also hypothesized that acetyl-CoA generated by nuclear PDC might be useful for lipid synthesis (de Boer and Houten, 2014). In this regard, we might also speculate that, in addition to histone acetylation, it is possible that increased acetyl-CoA generation by TUDCA-induced PDC in the NSC nucleus may also be required for lipid synthesis. Notably, this novel metabolic crosstalk triggered by TUDCA led to the acetylation levels of H3 that accompanied the increase in total H3 levels, possibly due to a nuclear functional PDC, which in turn might assure NSC cycle progression and differentiation-related epigenetic alterations. Finally, it would also be interesting to explore in the future possible changes of histone acetylation in the LCAD gene upon TUDCA treatment.

This study improves our understanding of how NSCs orchestrate adult neurogenesis in a metabolic-dependent manner, suggesting the TUDCA molecule as an efficient metabolic regulator for the maintenance and expansion of neural cells. This information together with the fact that the non-conjugated form of TUDCA is a FDA-approved molecule widely used in liver diseases and is currently in clinical trials for amyotrophic lateral sclerosis (Parry et al., 2010), provides a new framework to keep exploring its use in NSC maintenance and expansion. More importantly, we are now capable of moving toward a more comprehensive understanding of the metabolic machinery linking the processes of NSC proliferation and neuronal differentiation aimed at a more efficient use of neural replacement strategies.

## Data Availability Statement

The mass spectrometry proteomics data have been deposited to the ProteomeXchange Consortium via the PRIDE partner repository with the data set identifier PXD017979.

## Ethics Statement

Human and Animal Rights Sprague-Dawley rats were acquired from Charles River (France). All experimental procedures were in accordance with current Portuguese laws on Animal Care and with the European Union Directive (86/609/EEC; 2010/63/EU; 2012/707/EU), on the protection of animals used for experimental and other scientific purposes. All efforts were made to minimize animal suffering and reduce numbers.

## Author Contributions

MF was involved in the majority of the experiments and wrote the first version of the manuscript. MC and MR helped in cell culture and performed several experiments of Western blot and immunohistochemistry. SSi evaluated the ACC1 expression while SSS performed the immunoblotting and immunocytochemistry assays. JM and AC performed the proteomic assays and analyzed the data. MS performed the GC-MS analysis of fatty acids and analyzed the data. CR critically reviewed the manuscript and helped SSo in overseeing aspects related with TU. SSo conceived and designed the study, analyzed the data, and reviewed the manuscript. All authors wrote and approved the manuscript for publication.

## Conflict of Interest

The authors declare that the research was conducted in the absence of any commercial or financial relationships that could be construed as a potential conflict of interest.

## Supplementary Material

FIGURE S1 Early-differentiating NSCs were incubated with TUDCA for 24 h and collected for Western blot, as described in Materials and Methods. **(A)** Total levels of LCAD 24 h after siRNA transfection in NSCs. **(B)** Immunoblots showing the purity of the mitochondria and nuclei extracts of NSCs. **(C)** Total protein profile of histone purified extracts in NSCs.

TABLE S1 Identification of mitochondrial proteins differently expressed by TUDCA in mouse NSCs using LC-MS.

## References

[B1] Amaral J. D. Viana R. J. S. Ramalho R. M. Steer C. J. Rodrigues C. M. (2009). Bile acids: regulation of apoptosis by ursodeoxycholic acid. *J. Lipid Res.* 50 1721–1734. 10.1194/jlr.R900011-JLR200 19417220 PMC2724780

[B2] Bieberich E. (2012). It’s a lipid’s world: bioactive lipid metabolism and signaling in neural stem cell differentiation. *Neurochem. Res.* 37 1208–1229. 10.1007/s11064-011-0698-5 22246226 PMC3343224

[B3] Casarosa S. Bozzi Y. Conti L. (2014). Neural stem cells: ready for therapeutic applications? *Mol. Cell Ther.* 2:31. 10.1186/2052-8426-2-31 26056597 PMC4452059

[B4] Castro R. E. Solá S. Ma X. Ramalho R. M. Kren B. T. Steer C. J. (2005). A distinct microarray gene expression profile in primary rat hepatocytes incubated with ursodeoxycholic acid. *J. Hepatol.* 42 897–906. 10.1016/j.jhep.2005.01.026 15885361

[B5] Chegary M. Brinke H. T. Ruiter J. P. Wijburg F. A. Stoll M. S. Minkler P. E. (2009). Mitochondrial long chain fatty acid β-oxidation in man and mouse. *Biochim. Biophys. Acta* 1791 806–815. 10.1016/j.bbalip.2009.05.006 19465148 PMC2763615

[B6] Conti L. Pollard S. M. Gorba T. Reitano E. Toselli M. Biella G. (2005). Niche-independent symmetrical self-renewal of a mammalian tissue stem cell. *PLoS Biol.* 3:e283. 10.1371/journal.pbio.0030283 16086633 PMC1184591

[B7] Costa C. G. Dorland L. Holwerda U. de Almeida I. T. Poll-The B. T. Jakobs C. (1998). Simultaneous analysis of plasma free fatty acids and their 3-hydroxy analogs in fatty acid beta-oxidation disorders. *Clin. Chem.* 44 463–471. 9510849

[B8] Danbolt N. C. (2001). Glutamate uptake. *Prog. Neurobiol.* 65 1–105. 11369436 10.1016/s0301-0082(00)00067-8

[B9] de Boer V. C. Houten S. M. (2014). A mitochondrial expatriate: nuclear pyruvate dehydrogenase. *Cell* 158 9–10. 10.1016/j.cell.2014.06.018 24995972

[B10] Eberlé D. Hegarty B. Bossard P. Ferré P. Foufelle F. (2004). SREBP transcription factors: master regulators of lipid homeostasis. *Biochimie* 86 839–848. 10.1016/j.biochi.2004.09.018 15589694

[B11] Fan J. Lin R. Xia S. (2016). Tetrameric acetyl-CoA acetyltransferase 1 is important for tumor growth. *Mol. Cell.* 64 859–874. 10.1016/j.molcel.2016.10.014 27867011 PMC5135630

[B12] Fidaleo M. Cavallucci V. Pani G. (2017). Nutrients, neurogenesis and brain ageing: from disease mechanisms to therapeutic opportunities. *Biochem. Pharmacol.* 141 30283–30286. 10.1016/j.bcp.2017.05.016 28539263

[B13] Folmes C. D. L. Park S. Terzic A. (2013). Lipid metabolism greases the stem cell engine. *Cell Metab.* 17 153–155. 10.1016/j.cmet.2013.01.010 23395162

[B14] Fukao T. Yamaguchi S. Nagasawa H. Kano M. Orii T. Fujiki Y. (1990). Molecular cloning of cDNA for human mitochondrial acetoacetyl CoA thiolase and molecular analysis of 3-ketothiolase deficiency. *J. Inherit. Metab. Dis.* 13 757–760. 10.1007/bf01799582 1978869

[B15] Fukumoto S. Fujimoto T. (2002). Deformation of lipid droplets in fixed samples. *Histochem. Cell Biol.* 118 423–428. 10.1007/s00418-002-0462-7 12432454

[B16] Ginguay A. Cynober L. Curis E. Nicolis I. (2017). Ornithine Aminotransferase, an important glutamate-metabolizing enzyme at the crossroads of multiple metabolic pathways. *Biology* 6:E18. 10.3390/biology6010018 28272331 PMC5372011

[B17] Glaser T. Pollard S. M. Smith A. Brüstle O. (2007). Tripotential differentiation of adherently expandable neural stem (NS) cells. *PLoS One* 2:e298. 10.1371/journal.pone.0000298 17356704 PMC1808430

[B18] Gronbeck K. R. Rodrigues C. M. P. Mahmoudi J. Bershad E. M. Ling G. Bachour S. P. (2016). Application of Tauroursodeoxycholic acid for treatment of neurological and non-neurological diseases: is there a potential for treating traumatic brain injury? *Neurocrit. Care* 25 153–166. 10.1007/s12028-015-0225-7 26759227

[B19] Guo Q. Shi Q. Li H. Liu J. Wu S. Sun H. (2015). Glycolipid metabolism disorder in the liver of obese mice is improved by TUDCA via the restoration of defective hepatic autophagy. *Int. J. Endocrinol.* 2015:687938. 10.1155/2015/687938 26681941 PMC4668323

[B20] Hamilton L. K. Dufresne M. Joppé S. E. (2015). Aberrant lipid metabolism in the forebrain niche suppresses adult neural stem cell proliferation in an animal model of Alzheimer’s disease. *Cell Stem Cell* 17 397–411. 10.1016/j.stem.2015.08.001 26321199

[B21] He M. Rutledge S. L. Kelly D. R. Palmer C. A. Murdoch G. Majumder N. (2007). A New Genetic Disorder in Mitochondrial Fatty Acid β-Oxidation: ACAD9 Deficiency. *Am. J. Hum. Genet.* 81 87–103. 10.1086/519219 17564966 PMC1950923

[B22] Hertz L. Zielke H. R. (2004). Astrocytic control of glutamatergic activity: astrocytes as stars of the show. *Trends Neurosci.* 27 735–743. 10.1016/j.tins.2004.10.008 15541514

[B23] Houten S. M. Violante S. Ventura F. V. Wanders R. J. (2016). The biochemistry and physiology of Mitochondrial fatty acid β-oxidation and its Genetic disorders. *Annu. Rev. Physiol.* 78 23–44. 10.1146/annurev-physiol-021115-105045 26474213

[B24] Hu C. Fan L. Cen P. Chen E. Jiang Z. Li L. (2016). Energy metabolism plays a critical role in stem cell maintenance and differentiation. *Int. J. Mol. Sci.* 17:253. 10.3390/ijms17020253 26901195 PMC4783982

[B25] Jitrapakdee S. St Maurice M. Rayment I. Cleland W. W. Wallace J. C. Attwood P. V. (2008). Structure, mechanism and regulation of pyruvate carboxylase. *Biochem. J.* 413 369–387. 10.1042/BJ20080709 18613815 PMC2859305

[B26] Kann O. Kovács R. (2007). Mitochondria and neuronal activity. *Am. J. Physiol. Cell Physiol.* 292 C641–C657. 17092996 10.1152/ajpcell.00222.2006

[B27] Keene D. C. Rodrigues C. M. Eich T. Chhabra M. S. Steer C. J. Low W. C. (2002). Tauroursodeoxycholic acid, a bile acid, is neuroprotective in a transgenic animal model of Huntington’s disease. *Proc. Natl. Acad. Sci. U.S.A.* 99 10671–10676. 10.1073/pnas.162362299 12149470 PMC125009

[B28] Khacho M. Clark A. Svoboda D. S. Azzi J. MacLaurin J. G. Meghaizel C. (2016). Mitochondrial dynamics impacts stem cell identity and fate decisions by regulating a nuclear transcriptional program. *Cell Stem Cell* 19 232–247. 10.1016/j.stem.2016.04.015 27237737

[B29] Khacho M. Clark A. Svoboda D. S. MacLaurin J. G. Lagace D. C. Park D. S. (2017). Mitochondrial dysfunction underlies cognitive defects as a result of neural stem cell depletion and impaired neurogenesis. *Hum. Mol. Genet.* 26 3327–3341. 10.1093/hmg/ddx217 28595361 PMC5886206

[B30] Kiilerich K. Adser H. Jakobsen A. H. Pedersen P. A. Hardie D. G. Wojtaszewski J. F. (2010). PGC-1α increases PDH content but does not change acute PDH regulation in mouse skeletal muscle. *Am. J. Physiol. Regul. Integr. Comp. Physiol.* 299 1350–1359. 10.1152/ajpregu.00400.2010 20720174

[B31] Knobloch M. (2016). The role of lipid metabolism for neural stem cell regulation. *Brain Plast* 3 61–71. 10.3233/BPL-160035 PMC5928532 29765860

[B32] Knobloch M. Braun S. M. Zurkirchen L. von Schoultz C. Zamboni N. Araúzo-Bravo M. J. (2012). Metabolic control of adult neural stem cell activity by Fasn-dependent lipogenesis. *Nature* 493 226–230. 10.1038/nature11689 23201681 PMC3587167

[B33] Knobloch M. Jessberger S. (2017). Metabolism and neurogenesis. *Curr. Opin. Neurobiol.* 42 45–52. 10.1016/j.conb.2016.11.006 27915086

[B34] Knobloch M. Pilz G. A. Ghesquière B. Kovacs W. J. Wegleiter T. Moore D. L. (2017). A fatty acid oxidation-dependent metabolic shift regulates adult neural stem cell activity. *Cell Rep.* 20 2144–2155. 10.1016/j.celrep.2017.08.029 28854364 PMC5583518

[B35] Kompare M. Rizzo W. B. (2008). Mitochondrial fatty-acid oxidation disorders. *Semin. Pediatr. Neurol.* 15 140–149. 10.1016/j.spen.2008.05.008 18708005

[B36] Kuhn H. G. Dickinson-Anson H. Gage F. H. (1996). Neurogenesis in the Dentate Gyrus of the Adult decrease of neuronal progenitor proliferation. *J. Neurosci.* 16 2027–2033. 10.1523/JNEUROSCI.16-06-02027.1996 8604047 PMC6578509

[B37] Lin Z. Liu F. Shi P. Song A. Huang Z. Zou D. (2018). Fatty acid oxidation promotes reprogramming by enhancing oxidative phosphorylation and inhibiting protein kinase C. *Stem Cell Res. Ther.* 9:47. 10.1186/s13287-018-0792-6 29482657 PMC5937047

[B38] Lionaki E. Gkikas I. Tavernarakis N. (2016). Differential protein distribution between the nucleus and mitochondria: implications in aging. *Front. Genet.* 7:162. 10.3389/fgene.2016.00162 27695477 PMC5025450

[B39] Lisowski P. Kannan P. Mlody B. Prigione A. (2018). Mitochondria and the dynamic control of stem cell homeostasis. *EMBO Rep.* 19:e45432. 10.15252/embr.201745432 29661859 PMC5934764

[B40] Liu X. Cooper D. E. Cluntun A. A. Warmoes M. O. Zhao S. Reid M. A. (2018). Acetate production from glucose and coupling to mitochondrial metabolism in mammals. *Cell* 175 502–513. 10.1016/j.cell.2018.08.040 30245009 PMC6173642

[B41] Llombart V. García-Berrocosoa T. Bech-Serra J. J. (1996). Characterization of secretomes from a human blood brain barrier endothelial cells in-vitro model after ischemia by stable isotope labeling with aminoacids in cell culture (SILAC). *J. Proteomics* 133 100–112. 10.1016/j.jprot.2015.12.011 26718731

[B42] Ma D. K. Bonaguidi M. A. Ming G. L. Song H. (2009). Adult neural stem cells in the mammalian central nervous system. *Cell Res.* 19 672–682. 10.1038/cr.2009.56 19436263 PMC2738865

[B43] Mountford P. Zevnik B. Düwel A. Nichols J. Li M. Dani C. (1994). Dicistronic targeting constructs: reporters and modifiers of mammalian gene expression. *Proc. Natl. Acad. Sci. U.S.A.* 91 4303–4307. 10.1073/pnas.91.10.4303 8183905 PMC43773

[B44] Nichols J. Evans E. P. Smith A. G. (1990). Establishment of germ-line-competent embryonic stem (ES) cells using differentiation inhibiting activity. *Development* 110 1341–1348. 2129226 10.1242/dev.110.4.1341

[B45] Nunes A. F. Amaral J. D. Lo A. C. Fonseca M. B. Viana R. J. Callaerts-Vegh Z. (2012). TUDCA, a bile acid, attenuates amyloid precursor protein processing and amyloid-β deposition in APP/PS1 mice. *Mol. Neurobiol.* 45 440–454. 10.1007/s12035-012-8256-y 22438081

[B46] Ottoboni L. Merlini A. Martino G. (2017). Neural stem cell plasticity: advantages in therapy for the injured central nervous system. *Front. Cell Dev. Biol.* 5:52. 10.3389/fcell.2017.00052 28553634 PMC5427132

[B47] Ozcan U. Yilmaz E. Ozcan L. Ozcan U. Yilmaz E. Ozcan L. (2006). Chemical chaperones reduce ER stress and restore glucose homeostasis in a mouse model of type 2 diabetes. *Science* 313 1137–1140. 10.1126/science.1128294 16931765 PMC4741373

[B48] Park H. R. Kim J. Park K. Lee J. (2011). Lipotoxicity of palmitic acid on neural progenitor cells and hippocampal neurogenesis. *Toxicol. Res.* 27 103–110. 10.5487/TR.2011.27.2.103 24278558 PMC3834368

[B49] Parry G. J. Rodrigues C. M. Aranha M. M. (2010). Safety, tolerability, and cerebrospinal fluid penetration of ursodeoxycholic acid in patients with amyotrophic lateral sclerosis. *Clin. Neuropharmacol.* 33 17–21. 10.1097/WNF.0b013e3181c47569 19935406

[B50] Pollard S. M. Conti L. Sun Y. Goffredo D. Smith A. (2006). Adherent neural stem (NS) cells from fetal and adult forebrain. *Cereb. Cortex* 1 i112–i120. 10.1093/cercor/bhj167 16766697

[B51] Pratt T. Sharp L. Nichols J. Price D. J. Mason J. O. (2000). Embryonic stem cells and transgenic mice ubiquitously expressing a tau-tagged green fluorescent protein. *Dev. Biol.* 228 19–28. 10.1006/dbio.2000.9935 11087623

[B52] Rodrigues C. M. Fan G. Ma X. Kren B. T. Steer C. J. (1998a). A novel role for ursodeoxycholic acid in inhibiting apoptosis by modulating mitochondrial membrane perturbation. *J. Clin. Invest.* 101 2790–2799. 10.1172/JCI1325 9637713 PMC508870

[B53] Rodrigues C. M. Fan G. Wong P. Y. Kren B. T. Steer C. J. (1998b). Ursodeoxycholic acid may inhibit deoxycholic acid-induced apoptosis by modulating mitochondrial transmembrane potential and reactive oxygen species production. *Mol. Med.* 4 165–178. 9562975 PMC2230355

[B54] Rodrigues C. M. Keene C. D. Linehan-Stieers C. Ma X. Kren B. T. Low W. C. (2000). Tauroursodeoxycholic acid prevents apoptosis induced by 3-nitropropionic acid: evidence for a mitochondrial-dependent pathway independent of the permeability transition. *J. Neurochem.* 7 2368–2379. 10.1046/j.1471-4159.2000.0752368.x 11080188

[B55] Rodrigues C. M. Ma X. Linehan-Stieers C. Fan G. Kren B. T. Steer C. J. (1999). Ursodeoxycholic acid prevents cytochrome c release in apoptosis by inhibiting mitochondrial membrane depolarization and channel formation. *Cell Death Differ.* 6 842–854. 10.1038/sj.cdd.4400560 10510466

[B56] Rodrigues C. M. Sola S. Nan Z. Castro R. E. Ribeiro P. S. Low W. C. (2003). Tauroursodeoxycholic acid reduces apoptosis and protects against neurological injury after acute hemorrhagic stroke in rats. *Proc. Natl. Acad. Sci. U.S.A.* 100 6087–6092. 10.1073/pnas.1031632100 12721362 PMC156330

[B57] Schönfeld P. Reiser G. (2013). Why does brain metabolism not favor burning of fatty acids to provide energy? - Reflections on disadvantages of the use of free fatty acids as fuel for brain. *J. Cereb. Blood Flow Metab.* 33 1493–1499. 10.1038/jcbfm.2013.128 23921897 PMC3790936

[B58] Shi L. Tu B. P. (2015). Acetyl-CoA and the regulation of metabolism: mechanisms and Consequences. *Curr. Opin. Cell Biol.* 33 125–131. 10.1016/j.ceb.2015.02.003 25703630 PMC4380630

[B59] Shin J. Berg D. A. Zhu Y. (2015). Single-Cell RNA-Seq with waterfall reveals molecular cascades underlying adult neurogenesis. *Cell Stem Cell* 17 360–372. 10.1016/j.stem.2015.07.013 26299571 PMC8638014

[B60] Silva J. Chambers I. Pollard S. Smith A. (2006). Nanog promotes transfer of pluripotency after cell fusion. *Nature* 441 997–1001. 10.1038/nature04914 16791199

[B61] Smith A. G. (1991). Culture and differentiation of embryonic stem cells. *J. Tissue Cult. Methods* 13 89–94.

[B62] Soares R. Ribeiro F. F. Xapelli S. Genebra T. Ribeiro M. F. Sebastião A. M. (2018). Tauroursodeoxycholic acid enhances Mitochondrial biogenesis, neural stem cell pool, and early neurogenesis in adult rats. *Mol. Neurobiol. Mol. Neurobiol.* 55 3725–3738. 10.1007/s12035-017-0592-5 28534273

[B63] Sohur U. S. Emsley J. G. Mitchell B. D. Macklis J. D. (2006). Adult neurogenesis and cellular brain repair with neural progenitors, precursors and stem cells. *Philos. Trans. R. Soc. Lond. B Biol. Sci.* 361 1477–1497. 10.1098/rstb.2006.1887 16939970 PMC1664671

[B64] Spiliotopoulos D. Goffredo D. Conti L. Di Febo F. Biella G. Toselli M. (2009). An optimized experimental strategy for efficient conversion of embryonic stem (ES)-derived mouse neural stem (NS) cells into a nearly homogeneous mature neuronal population. *Neurobiol. Dis.* 34 320–331. 10.1016/j.nbd.2009.02.007 19236914

[B65] Sutendra G. Kinnaird A. Dromparis P. Paulin R. Stenson T. H. Haromy A. (2014). A nuclear pyruvate dehydrogenase complex is important for the generation of Acetyl-CoA and histone acetylation. *Cell* 158 84–97. 10.1016/j.cell.2014.04.046 24995980

[B66] Tang B. L. (2015). Mitochondrial protein in the nucleus. *Cell Bio* 4 23–29.

[B67] van Vlies N. Tian L. Overmars H. Bootsma A. H. Kulik W. Wanders R. J. (2005). Characterization of carnitine and fatty acid metabolism in the long-chain acyl-CoA dehydrogenase-deficient mouse. *Biochem. J.* 387(Pt 1), 185–193. 10.1042/BJ20041489 15535801 PMC1134946

[B68] Vang S. Longley K. Steer C. J. Low W. C. (2014). The unexpected uses of Urso- and Tauroursodeoxycholic acid in the treatment of Non-liver diseases. *Glob. Adv. Health Med.* 3 58–69. 10.7453/gahmj.2014.017 24891994 PMC4030606

[B69] Varum S. Rodrigues A. S. Moura M. B. Momcilovic O. Easley C. A. I. V. Ramalho-Santos J. (2011). Energy metabolism in human pluripotent stem cells and their differentiated counterparts. *PLoS One* 6:e20914. 10.1371/journal.pone.0020914 21698063 PMC3117868

[B70] Wallace D. C. (2005). A mitochondrial paradigm of metabolic and degenerative diseases, aging, and cancer: a dawn for evolutionary medicine. *Annu. Rev. Genet.* 39 359–407. 10.1146/annurev.genet.39.110304.095751 16285865 PMC2821041

[B71] Wanet A. Arnould T. Najimi M. Renard P. (2015). Connecting mitochondria. metabolism, and stem cell fate. *Stem Cells Dev.* 24 1957–1971. 10.1089/scd.2015.0117 26134242 PMC4543487

[B72] Wellen K. E. Hatzivassiliou G. Sachdeva U. M. Bui T. V. Cross J. R. Thompson C. B. (2009). ATP-citrate lyase links cellular metabolism to Histone Acetylation. *Science* 324 1076–1080. 10.1126/science.1164097 19461003 PMC2746744

[B73] Xavier J. M. Morgado A. L. Rodrigues C. M. Solá S. (2014). Tauroursodeoxycholic acid increases neural stem cell pool and neuronal conversion by regulating mitochondria-cell cycle retrograde signaling. *Cell Cycle* 13 3576–3589. 10.4161/15384101.2014.962951 25483094 PMC4613652

[B74] Xavier J. M. Rodrigues C. M. P. Solá S. (2015). Mitochondria: major regulators of neural development. *Neuroscientist* 22 346–358. 10.1177/1073858415585472 25948649

[B75] Zhang H. (2015). HIF-1 suppresses lipid catabolism to promote cancer progression. *Mol. Cell Oncol.* 2:e980184 10.4161/23723556.2014.980184 PMC4905416 27308514

[B76] Zhang Y. Bharathi S. S. Beck M. E. Goetzman E. S. (2019). The fatty acid oxidation enzyme long-chain acyl-CoA dehydrogenase can be a source of mitochondrial hydrogen peroxide. *Redox Biol.* 26:101253. 10.1016/j.redox.2019.101253 31234015 PMC6597861

[B77] Ziegler A. N. Levison S. W. Wood T. L. (2015). Insulin and IGF receptor signalling in neural stem-cell homeostasis. *Nat. Rev. Endocrinol.* 11 161–170. 10.1038/nrendo.2014.208 25445849 PMC5513669

