# Supplementary material for: Reprogramming of Lipid Metabolism as a New Driving Force Behind Tauroursodeoxycholic Acid-Induced Neural Stem Cell Proliferation
Source: Front Cell Dev Biol. 2020 May 26;8:335. doi: 10.3389/fcell.2020.00335 (PMC7286385; doi:10.3389/fcell.2020.00335)

Supplementary table 1

Protein	Uniprot Accession Code	Identification Score*	Number of unique peptides	Sequence Coverage (%)
Long-chain acyl-CoA dehydrogenase (LCAD)	P51174	323	16	36
Ornithine aminotransferase (OAT)	Q3UKT3	215	20	49
Aldehyde dehydrogenase 2 (ALDH2)	Q544B1	178	16	32
Acetyl-CoA acetyltransferase (ACAT1)	Q8QZT1	323	16	44

*Identification score was calculated by MaxQuant and it is derived from peptide posterior error probabilities.

Supplementary figure S1

S1A

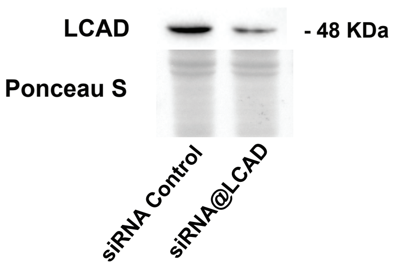

S1B

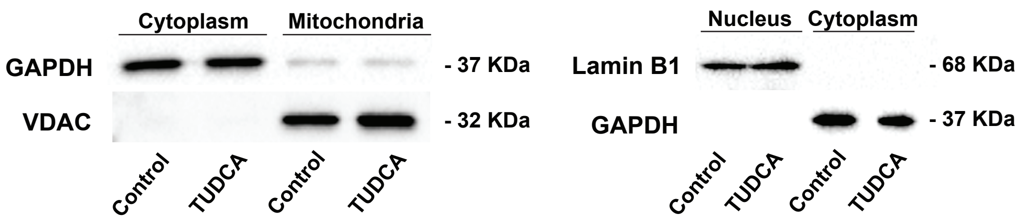

S1C

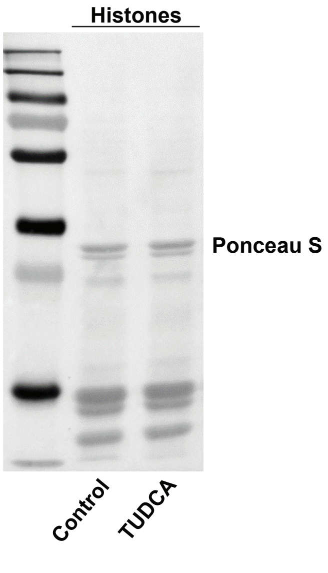

Supplement: FIGURE S1 — Early-differentiating NSCs were incubated with TUDCA for 24 h and collected for Western blot, as described in Materials and Methods. (A) Total levels of LCAD 24 h after siRNA transfection in NSCs. (B) Immunoblots showing the purity of the mitochondria and nuclei extracts of NSCs. (C) Total protein profile of histone purified extracts in NSCs. [file Data_Sheet_1.pdf]
